# Burden of lung cancer and application of patient-reported outcomes in the Western Pacific Region: a systematic analysis

**DOI:** 10.3389/fmed.2025.1700961

**Published:** 2025-12-31

**Authors:** Kejia Liu, Qiantai Gao, Xiumei Wang, Jian Lan, Tian Tian, Hongguo Rong

**Affiliations:** 1 Center for Evidence-Based Chinese Medicine, Beijing University of Chinese Medicine, Beijing, China; 2 Key Laboratory of Chinese Internal Medicine of Ministry of Education, Dongzhimen Hospital, Beijing University of Chinese Medicine, Beijing, China; 3 School of Humanities, Beijing University of Chinese Medicine, Beijing, China; 4 School of Traditional Chinese Medicine, Beijing University of Chinese Medicine, Beijing, China; 5 Institute for Excellence in Evidence-Based Chinese Medicine, Beijing University of Chinese Medicine, Beijing, China

**Keywords:** Global Burden of Disease, lung cancer, patient-reported outcomes, quality of life, Western Pacific Region

## Abstract

**Background:**

The burden of lung cancer and the experiences of patients necessitate attention. This study evaluated the disease burden of lung cancer and the attributes of patient-reported outcomes (PROs) in clinical trials pertaining to lung cancer within the Western Pacific Region (WPRO).

**Methods:**

Drawing upon data from the Global Burden of Disease Study (GBD) 2021, the incidence, mortality, and disability-adjusted life years in the WPRO from 1990 to 2021 was retrieved and analyzed. Clinical trial data conducted within the WPRO spanning from 2010 to 2022 were extracted from International Clinical Trials Registry Platform, encompassing trial phases, study settings, participant demographics, and PRO instruments. Trials were categorized into three groups based on the mention of PRO usage and the utilization of specified PRO instruments.

**Results:**

In 2021, the WPRO witnessed 1.15 million (95% UI 0.96–1.36) new lung cancer cases and 0.99 million (95% UI 0.82–1.16) deaths. From 1990 to 2021, Singapore showed the most pronounced decline in the age standardized incidence (−38.62, 95% UI −46.63 to −30.99), while China was experiencing the highest increase (32.92, 95% UI 1.21–71.31). Among the 3,889 trials examined, 24.48% trials employed PRO instruments as outcome measures. A majority of these PRO-related trials were conducted in China, Japan, Australia and South Korea, with frequently used PRO instruments including the European Organization for Research and Treatment of Cancer Quality of Life Questionnaire-Core 30, the European Organization for Research and Treatment of Cancer Quality of Life Questionnaires-Lung Cancer 13, and the Visual Analogue Scale.

**Conclusion:**

Marked disparities existed in the lung cancer burden among the individual countries in the WPRO. There is a pressing need to allocate further resources toward the implementation of standardized, condition-specific PROs and strategies aimed at minimizing the lung cancer burden, especially in low and low-middle SDI countries.

## Introduction

Lung cancer stands as the leading cause of cancer-related deaths and ranks the sixth among all causes of mortality globally (1–3). According to projections by the International Agency for Research on Cancer, 1.31 million fresh cases of lung cancer were documented in the Western Pacific Region (WPRO) in 2022, comprising 52.7% of the global totally (4).

Over the past three decades, the 5-year relative survival rate for lung cancer has witnessed a notable surge (5). As survivorship stretches, a heightened emphasis must be place on enhancing the quality of life of patients (6). In addition, lung cancer exacts a heavy personal toll, manifesting in severe symptoms such as cough, pain, fatigue, depression and other impairments (7, 8). Notably, the WPRO bears a disproportionately higher burden of lung cancer compared to other regions, yet research delving into this burden and the utilization of PROs in the WPRO remains scant (4).

The Global Burden of Disease, Injuries, and Risk Factors Study (GBD) 2021 provides estimates spanning incidence, mortality, prevalence and Disability-adjusted life years (DALYs) associated for 371 diseases and injuries along with 88 risk factors across 204 countries and territories from 1990 to 2021. Patient-reported outcomes (PROs), which encompass direct patient reports on their health status, including symptoms, treatment adherence, functioning, and health-related quality of life (9), are gathered through systematic and rigorous methodologies. PRO instruments are invaluable in capturing the impact of disease from the patient’s perspective (10, 11), ultimately enhancing patient-clinician communication, clinician awareness of symptoms, symptom management, patient satisfaction, and overall survival (12). Consequently, PRO instruments are widely adopted as outcomes in cancer clinical trials for their immense value (13, 14).

By analyzing data sourced from the GBD 2021 study, this study endeavor presents comprehensive estimates about lung cancer incidence, fatalities and DALYs in the WPRO, spanning from 1990 to 2021, and categorizes these estimates according to country, age, sex and socio-demographic index (SDI). Furthermore, it scrutinizes the incorporation of PROs in clinical trials conducted within the WPRO from 2010 to 2022 to evaluate the impact of PROs on alleviating the lung cancer burden.

## Methods

### Study design

This systematic analysis extracted the registration information of adult interventional cancer clinical trials focused on lung cancer in the WPRO, to examine the utilization of PROs. Additionally, it leverages publicly accessible estimates from the GBD 2021 study to explore the lung cancer burden, ultimately aiming to advocate for the adoption of standardized and condition-specific PRO instruments to diminish the lung cancer burden.

### Data collection strategy

This study extracted annual crude and age-standardized estimates of different measures of the lung cancer burden metrics from the GBD 2021 database from 1990 to 2021, inclusive of their respective 95% uncertainty intervals (UIs) via the Global Health Data Exchange query tool. The data encompasses prevalent cases, incidence rates, mortality counts, number of disability-adjusted life-years and their corresponding age-standardized rates (ASRs) within WPRO. The countries, territories, and areas constituting the WPRO are listed in Multimedia Appendix 1.

**Figure 1:**
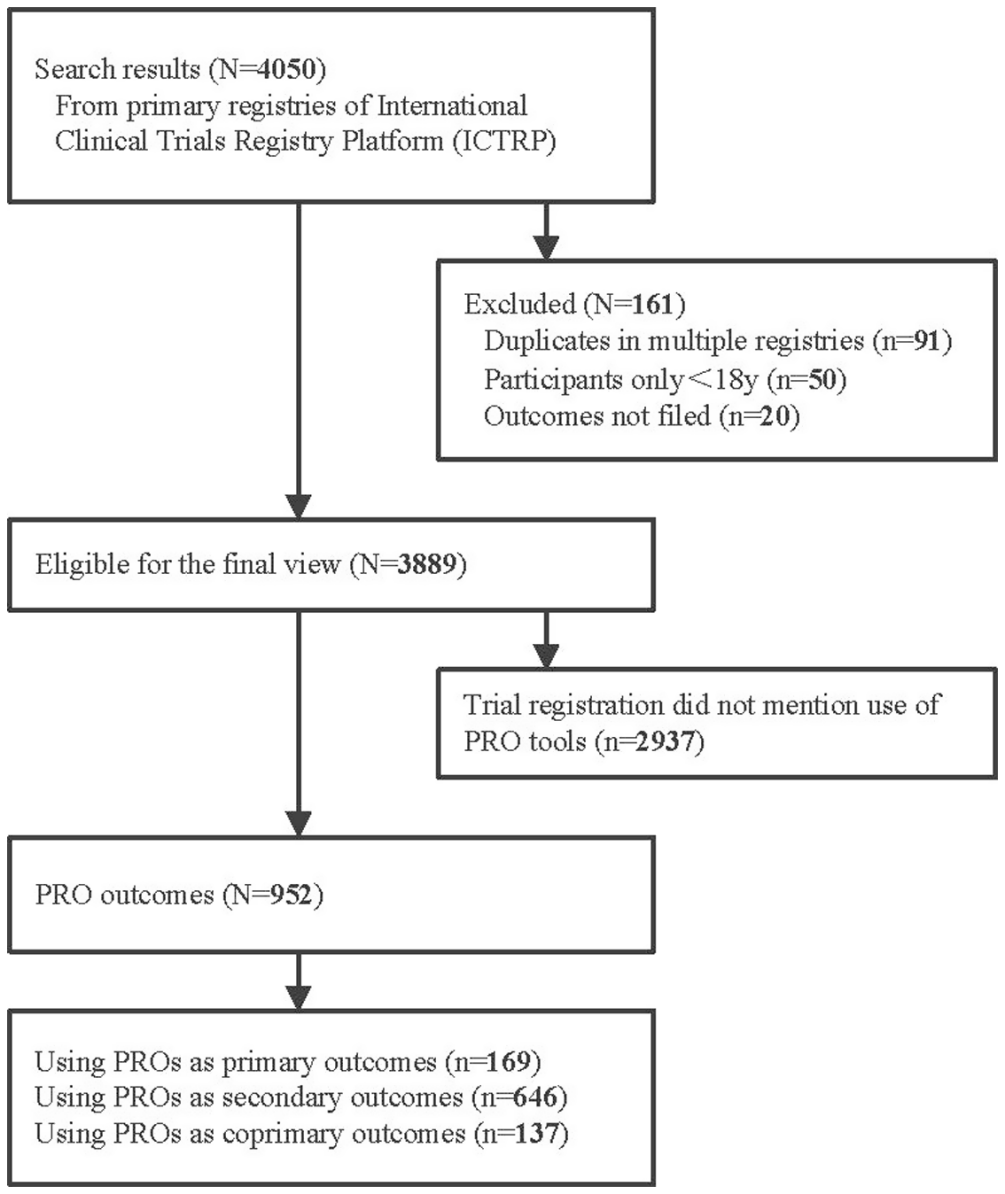
Trial exclusion and classification criteria. PRO: patient-reported outcome.

Data from clinical trials conducted in the WPRO was retrieved from International Clinical Trials Registry Platform. For each included trial, a comprehensive array of data was extracted, including trial phase, participant demographics, randomization procedures, blinding, recruitment status and the PRO instruments utilized (Searching Strategy in Multimedia Appendix 1). This study was aimed at assessing the defining features of lung cancer between January 1, 2010 and December 31, 2022. A total of 4,050 lung cancer clinical trials were considered eligible, with 161 trials being excluded due to duplicates across multiple registries, inclusion of participants under 18 years of age, or missing or unreported outcomes. The identification of trials is depicted in the Figure 1. The information of the clinical trials was as follows: Basic information, containing the number of registrations, registration date, official title and countries involved. Key information, including PRO instruments, target size, the age and gender of participants. Characteristic attributes, like the location of the primary sponsor, study phase and the interventional model adopted.

### Data analysis

Age-standardized rate was used to estimate prevalence, incidence, mortality and DALYs associated with the lung cancer, with 95% UIs provided for each estimate. The Socio-demographic Index integrates comprehensive data on economic status, educational attainment, and fertility rates across countries, exemplifying their socio-economic development.

The clinical trials were categorized into three groups: those that explicitly mentioned PRO usage and the identified specific PRO instruments, those that mentioned PRO usage without specifying the instruments, and those that did not mention PRO usage in their registration information. PRO measures were identified from the PROQOLID database, as it specifies whether an instrument is a PRO measure. KL and QG independently extracted detailed characteristics of the included trials using a pre-designed data extraction table, encompassing the study phase, participant demographics, interventional model, recruitment status, and represented geographical regions. In cases of disagreement, a third author XW served as the arbiter to make a final determination. Given the potential overlap PRO instrument for both primary and secondary endpoints within trials, this study consolidated the PRO instruments used across each trial to ascertain the most prevalent measures. Quantitative analyses were conducted solely on items that specified the names of PRO instruments, aiming to provide a comprehensive overview of the commonly utilized evaluation tools. These analyses were executed using R 4.3.2.

### Ethical considerations

This study is based on the secondary analysis of data derived from the 2021 GBD study, which is an aggregation and anonymization of data sets. According to the Common Rule (45 CFR part 46) of the US Department of Health and Human Services (Office for Human Research Protections), this study is exempt from institutional review board approval and the requirement for informed consent because it did not involve clinical data or human participants. Thus, this study did not necessitate a separate institutional review board approval or exemption.

## Results

### Regional trend

In 2021, the WPRO faced a substantial burden of lung cancer, with 1.15 million (95% UI 0.96–1.36) new cases, 0.99 million deaths (95% UI 0.82–1.16), and 2.24 million DALYs (95% UI 1.84–2.66). The age-standardized incidence rate (ASIR) for both sexes combined was 40.17 (95% UI 33.47–47.36), while the ASR for mortality and DALYs was 34.70 deaths per 100,000 population (95% UI 28.80–40.78) and 782.12 per 100,000 population (95% UI 643.87–927.79), respectively (Supplementary Table 1).

Notably, the disease burden of lung cancer in the WPRO showed a clear upward trend from 1990 to 2021. The ASIR increased by 26.50% (95% UI 0.89–55.46%) over the period, with a more pronounced rise in females (38.19, 95% UI 7.56–75.81) compared to males (20.33, 95% UI 11.99–56.26). In contrast, the age-standardized mortality rate (ASMR) grew more moderately, with 4.46% (95% UI −24.04–36.20) in males and 16.96% (95% UI −8.84–48.58) in females.

### Regional trends by age and sex groups

**Figure 2:**
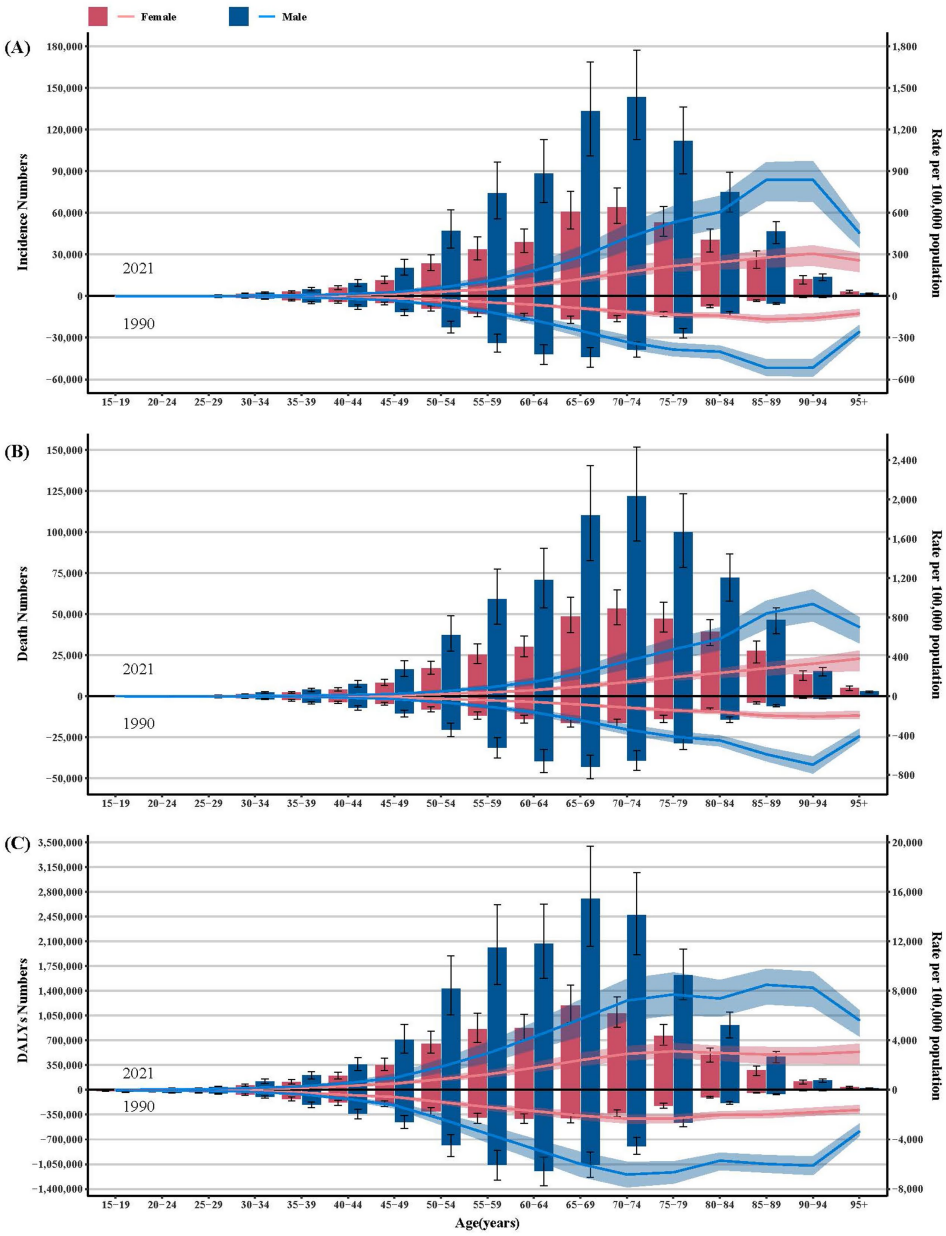
The number and age-standardized rate of incidence **(A)**, deaths **(B)**, and DALYs **(C)** of lung cancer in the Western Pacific Region. Shade area indicates 95% upper and lower uncertainty intervals, respectively.

Figure 2 shows the incidence, mortality and DALYs due to lung cancer in 1990 and 2021, stratified by age and sex, along with their respective ASRs. For both genders, the incidence and mortality counts peaked in the 70–74 age group in 2021 and the 65–69 age group in 1990. DALYs peaked in the 65–69 age group in 2021 and the 60–64 age bracket in 1990 for both sexes. The highest ASIR was observed among individuals aged 90–94 years in 2021 and 85–89 years in 1990 for both genders. The ASMR reached its zenith in the 90–94 age group for males and 95 + age group for females in both time periods. The age-standardized DALYs rate (ASDR) peaked in the 85–89 age group for males and the 75–79 age group for females in 2021, whereas in 1990 it peaked in the 70–74 age group for both genders. Within the 15–19, 20–24 and 25–29 age groups, a notable decline was observed in these metrics and their ASRs in 2021 relative to 1990.

### National trend

Marked differences in lung cancer burden emerged across WPRO countries in 2021. For ASIR, Palau led the list at 44.74 (95% UI l 35.53–55.18) per 100,000 population, closely followed by China (44.01, 95% UI 35.45–53.35). In contrast, Samoa had the lowest ASIRs (8.28, 95% UI 6.22–10.62). Similar extremes were observed for mortality. Palau also had the highest ASMR (48.27, 95% UI 38.23–59.49), while Singapore recorded the lowest (16.19, 95% UI 14.52–18.03).

From 1990 to 2021, national trends diverged sharply. China saw the most dramatic increases in ASIR (32.92, 95% UI 1.21–71.31%). On the opposite, Singapore experienced the steepest ASIR declines, at 38.6 and 31.3%. Singapore also stood out for the largest reductions in both ASMR (−52.98, 95% UI −58.73% to −47.22%) (Supplementary Table 1).

### National burden of lung cancer by the socio-demographic index

Supplementary Figure 1 reveals that the burden of lung cancer among 27 countries in the WPRO did not exhibit a clear correlation with the SDI. However, an intriguing pattern emerged that until an SDI level of 0.4, a downward trend was observed in the ASRs of incidence, mortality, and DALYs. Subsequently, these rates ascended, peaking at an SDI level of 0.7, before declining again across the remaining SDI levels. Moreover, considerable heterogeneity was evident in the lung cancer burden among countries with SDI level ranged from 0.45 to 0.75. Some countries, such as Samoa, Fiji, remained well below expected levels throughout the study period, with minimal changes in their ASRs. In contrast, others, like Mongolia, China, and Palau, were above expected levels but experienced fluctuating or decreasing ASRs.

### Clinical trial characteristics

A total of 4,050 interventional studies conducted in the WPRO were identified. Our study excluded 161 trials, including 91 duplicates, 50 clinical trials involving participants under 18 years old, and 20 trials with incomplete or unreported outcomes, thereby yielding 3,889 eligible trials for the final review. Among these 952 trials mentioned the use of PROs, including 169 trials using PROs as primary outcomes, 646 as secondary outcomes and 137 as coprimary outcomes. Additionally, 2,937 trials did not mention the use of PRO instruments.

Table 1 provides a comprehensive overview of eligible trials. Single arm trials (1971, 50.68%) comprised the largest proportion, followed by parallel trials (1736, 44.64%). Within PRO-related trials, parallel trials were predominant (676, 71.01%). Open-label trials constituted the highest proportion for both PRO-related trials and all included trials. Phase II trials were the most frequent among all trails (1,526, 39.24%) and PRO-related trials (249, 26.16%). However, the proportion of PRO application in Phase II trials (16.32%) was significantly lower than in Phase III (25.56%) and Phase IV (27.27%) trials. Virtually all subjects in both the overall (3,863, 99.33%) and PRO-specific (945, 99.26%) trials were adults. In terms of sex, 98.69% of all trials did not impose any restrictions. Among 959 PROs trials, 98.11% included both sexes. Regarding the use of PRO instruments, 257 PRO trials (27.00%) used only 1 specified PRO instrument, and 219 (23.00%) used 2 to 4 specified PRO instruments, 17 (1.79%) used 5 to 8 instruments, and 3(0.32%) used more than 9 instruments. Meanwhile, 456 trials (47.90%) did not use any specified PRO instruments.

**Table 1:** Characteristics of all identified trials and patient-reported outcome (PRO) related trials.

Characteristics	Trials, No. (%)	Trials using PROs, No. (%)
Total	3,889	952
Intervention model		
Single arm	1971 (50.68)	247 (25.95)
Parallel	1736 (44.64)	676 (71.01)
Crossover	27 (0.69)	5 (0.53)
Other^a^	108 (2.78)	16 (1.68)
Unclear	47 (1.21)	8 (0.84)
Blinding		
Open-label	2,699 (69.40)	443 (46.53)
Single blinded	102 (2.62)	52 (5.46)
Double blinded	203 (5.22)	89 (9.35)
Triple blinded	44 (1.13)	22 (2.31)
Quadruple blinded	88 (2.26)	26 (2.73)
Unclear	753 (19.36)	320 (33.61)
Phase		
0	322 (8.28)	133 (13.97)
I	407 (10.47)	69 (7.25)
II	1,526 (39.24)	249 (26.16)
III	399 (10.26)	102 (10.71)
IV	215 (5.53)	75 (7.88)
Other^b^	597 (15.35)	199 (20.90)
Unclear	405 (10.41)	125 (13.13)
Age (years)		
≥18	3,863 (99.33)	945 (99.26)
≥65	3 (0.08)	1 (0.11)
Unclear	23 (0.59)	6 (0.63)
Sex		
Male	42 (1.08)	13 (1.37)
Female	6 (0.15)	3 (0.32)
Both sexes	3,838 (98.69)	934 (98.11)
Unclear	3 (0.08)	2 (0.21)
Sample size		
0–200	3,273 (84.16)	747 (78.47)
201–400	338 (8.69)	13 (1.37)
401–1,000	220 (5.66)	61 (6.41)
≥1,000	51 (1.31)	13 (1.37)
Unclear	7 (0.18)	1 (0.11)
PRO instruments used		
1	N/A	257 (27.00)
02-April	N/A	219 (23.00)
05-August	N/A	17 (1.79)
≥9	N/A	3 (0.32)
Unspecified^c^	N/A	456 (47.90)

a Factorial, sequential assignment, and others.

b Diagnostic new technique clinical study, inspection technology, and trials involving multiple phases.

c Trials used unspecified PRO instruments could not be counted.

### Conditions and PRO instruments

**Figure 3:**
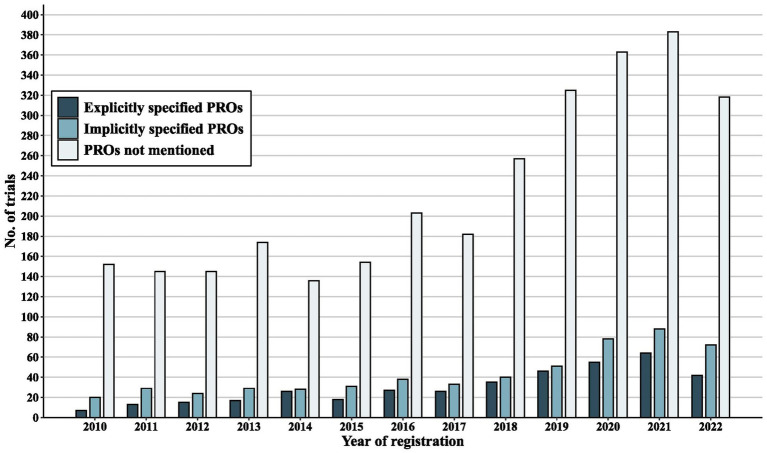
Annual number of clinical trials analyzed.

Figure 3 illustrates the increase in the annual counts of lung cancer clinical trials registered in the WPRO from 2010 to 2022, highlighting an increased proportion of trials that incorporated PRO instruments, with a particular emphasis on those that explicitly specified PRO instruments in their investigating lung cancer trials. Among the 3,889 trials deemed eligible for the definitive review, a notable 12.75% (*n* = 496) participated in trials that reported specified PRO instruments, 11.73% (*n* = 456) were involved in trials that reported unspecified PRO instruments, and a substantial 75.52% (*n* = 2,937) comprised trials that did not use PRO instruments.

Table 2 illustrates the frequency of PRO instruments utilized for lung cancer trials with specified PRO instruments across various outcomes. When PRO instruments served as primary outcomes, the Visual Analog Scale (VAS) (32, 32.99%) and the Numeric Rating Scale (NRS) (21, 21.65%) emerged as the most frequently employed scales. Additionally, in trials where PRO instruments were secondary outcomes, the European Organization for Research and Treatment of Cancer Quality of Life Questionnaires-Lung Cancer 13 (EORTC QLQ-LC13) (69, 24.21%) topped the list, followed by the Functional Assessment of Cancer Therapy-Lung (FACT-L) (38, 13.33%), European Quality of Life-5 Dimensions (EQ-5D) (30, 10.53%) and VAS (23, 8.07%). For trials incorporating PRO instruments as coprimary outcomes, the European Organization for Research and Treatment of Cancer Quality of Life Questionnaire-Core 30 (EORTC QLQ-C30) (34, 29.82%) was the most frequently used, with VAS (25, 7.36%), HADS (18, 15.79%) and NRS (17, 14.91%) also being commonly employed. Of all trails using PRO instruments, the EORTC QLQ-C30 (165, 33.27%) and EORTC QLQ-LC13 (84, 16.94%) l dominated the landscape, followed by VAS (80, 16.13%), FACT-L (53, 10.69%) and NRS (49, 9.88%).

**Table 2:** High-frequency of PRO instruments used in different outcomes.

No.	Primary outcome, No. (%)	Secondary outcome, No. (%)	Coprimary outcome, No. (%)	Total, No. (%)
1	VAS (32, 32.99%)	EORTC QLQ-LC13 (69, 24.21%)	EORTC QLQ-C30 (34, 29.82%)	EORTC QLQ-C30 (165, 33.27%)
	(Pain)	(QoL in lung cancer patients)	(QoL in lung cancer patients)	(QoL in lung cancer patients)
2	NRS (21, 21.65%)	FACT-L (38, 13.33%)	VAS (25, 7.36%)	EORTC QLQ-LC13 (84, 16.94%)
	(Pain)	(QoL in lung cancer patients)	(Pain)	(QoL in lung cancer patients)
3	EORTC QLQ-C30 (10, 10.31%)	EQ-5D (30, 10.53%)	HADS (18, 15.79%)	VAS (80, 16.13%)
	(QoL in lung cancer patients)	(QoL)	(Anxiety and depression)	(Pain)
4	FACT-L (7, 7.22%)	VAS (23, 8.07%)	NRS (17, 14.91%)	FACT-L (53, 10.69%)
	(QoL in lung cancer patients)	(Pain)	(Pain)	(QoL in lung cancer patients)
5	QoR-40 (5, 5.15%)	EORTC QLQ-C30 (19, 6.67%)	EORTC QLQ-LC13 (11, 9.65%)	NRS (49, 9.88%)
	(Quality of recovery after anesthesia)	(QoL in lung cancer patients)	(QoL in lung cancer patients)	(Pain)
6	EORTC QLQ-LC43 (4, 4.12%)	HADS (16, 5.61%)	BFI (9, 7.89%)	EQ-5D (42, 8.47%)
	(QoL in lung cancer patients)	(Anxiety and depression)	(Fatigue)	(QoL)
7	EORTC QLQ-LC13 (3, 3.09%)	LCSS (12, 4.21%)	EQ-5D (9, 7.89%)	HADS (37, 7.46%)
	(QoL in lung cancer patients)	(QoL in lung cancer patients)	(QoL)	(Anxiety and depression)
8	EQ-5D (3, 3.09%)	NRS (11, 3.86%)	FACT-L (8, 7.02%)	LCSS (14, 2.82%)
	(QoL)	(Pain)	(QoL in lung cancer patients)	(QoL in lung cancer patients)
9	HADS (3, 3.09%)	SF-36/SF-36v2 (9, 3.16%)	CFS (7, 6.14%)	BFI (12, 2.42%)
	(Anxiety and depression)	(QoL)	(Fatigue)	(Fatigue)
10	LCQ (3, 3.09%)	SGRQ (6, 2.11%)	PSQI (7, 6.14%)	SF-36 / SF-36v2 (12, 2.42%)
	(Cough)	(QoL in chronic airways diseases)	(Sleep quality)	(QoL)

BFI, Brief fatigue inventory; CFS, Cancer fatigue scale; EORTC QLQ-C30, European Organization for Research and Treatment of Cancer Quality of Life Questionnaire-Core 30; EORTC QLQ-LC13, European Organization for Research and Treatment of Cancer Quality of Life Questionnaires-Lung Cancer 13; EORTC QLQ-LC43, European Organization for Research and Treatment of Cancer Quality of Life Questionnaire-Lung Cancer 43; EQ-5D, European Quality of Life-5 Dimensions; FACT-L, Functional Assessment of Cancer Therapy-Lung; HADS, Hospital Anxiety Depression Scale; LCQ, Leicester Cough Questionnaire; LCSS, Lung Cancer Symptom Scale; NRS, Numeric Rating Scale; PSQI, Pittsburgh Sleep Quality Index; QoL, Quality of Life; QoR-40, Quality of Recovery-40; SF-36/SF-36v2, Short-Form 36-item Health Survey; SGRQ, St George’s Respiratory Questionnaire; VAS, Visual Analogue Scale.

**Figure 4:**
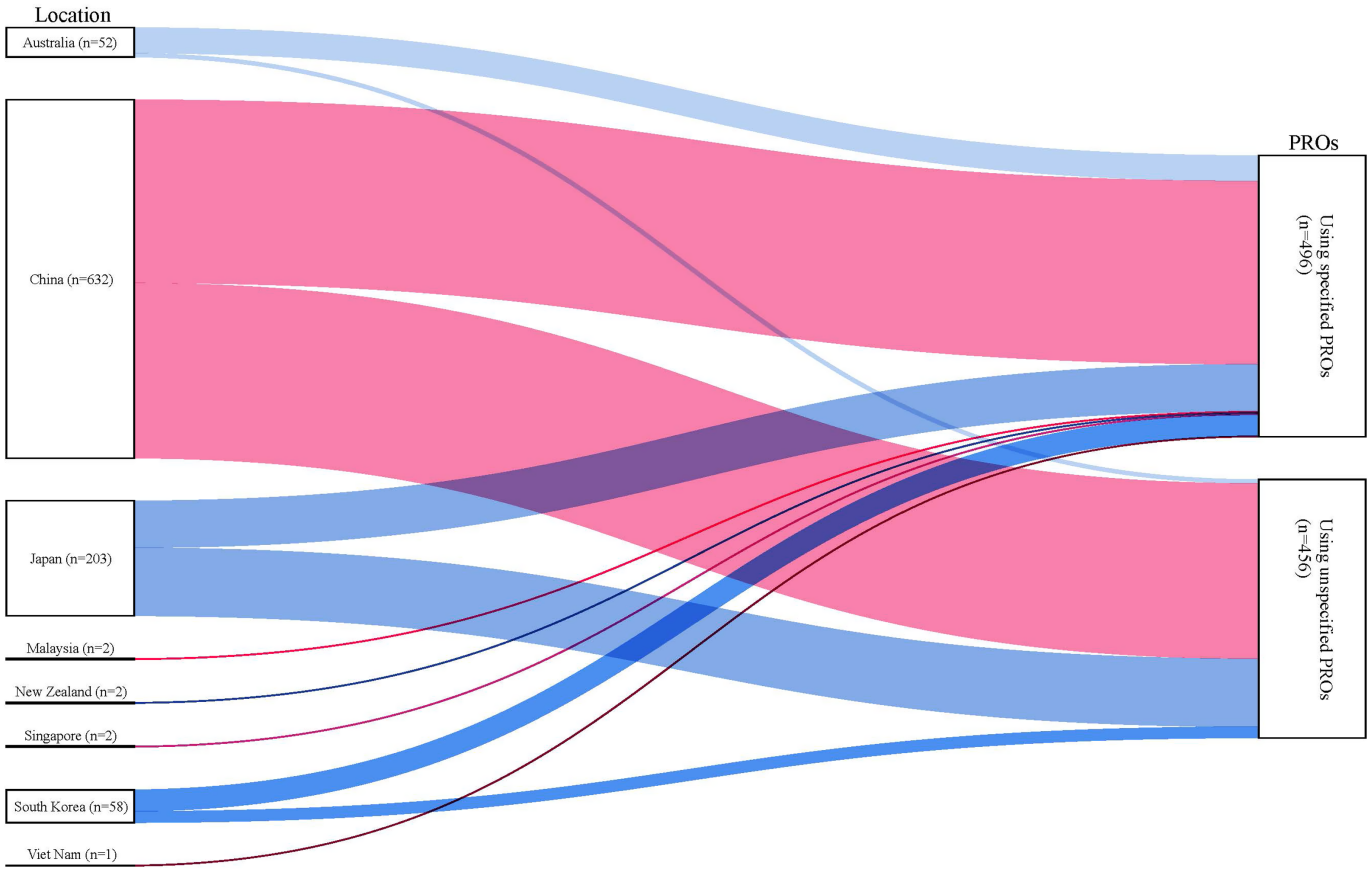
Distribution of PRO-related trials analyzed.

Figure 4 depicts the geographical distribution of PRO-related clinical trials in countries within the WPRO. It is noteworthy that the majority of countries in the WPRO have neglected to harness the potential of PRO tools for the evaluating clinical trials, with only a minority having conducted a substantial number of trials incorporating PROs. Specifically, China conducted 66.39% (632/952) of PRO-related trials, followed by Japan (21.32%, 203/952), South Korea (6.09%, 58/952) and Australia (5.46%, 52/952). Although China accounted for a considerable number of PRO-related trials, the proportion of specified PROs employed remained relatively low. Conversely, Australia and South Korea hosted a limited quantity of such trials, they demonstrated a relatively high proportion of specified PROs utilization.

## Discussion

This study evaluated the impact of lung cancer and the incorporation of PROs in clinical trials within the WPRO. The finding revealed the substantial burden of lung cancer coupled with the inadequate utilization of PROs in the WPRO, underscoring the imperative for widespread adoption of standardized and condition-specific PRO instruments to effectively alleviate this burden.

During the study period, WPRO witnessed an augmentation in the lung cancer burden, accompanied by a multifaceted disparity encompassing sex, age, and country-specific variations. From 1990 to 2021, the ASIR, AMSR and ASDR continued to climb for females. It is noteworthy that the lung cancer burden in the WPRO surpassed the global burden for both sexes, as previously reported (15). This study also revealed an age-dependent increase in lung cancer burden, with peak ASIR, ASMR and ASDR observed in those aged ≥80 years. The increased burden among older populations may stem from a combination of factors, including heightened screening rates, undertreatment and longer exposure to risk factors (5, 16). Moreover, males bore a heavier lung cancer burden than females, which could be attributed to their higher prevalence of risk factors such as smoking, dietary and occupational exposures (17), despite females demonstrated a heightened susceptibility to lung cancer upon smoking (18). It is recommended to improve screening effectiveness by targeting high-risk population and implementing personalized screening strategies (19). Notably, a decrease in the lung cancer incidence was observed among individuals aged under 30 years, potentially due to the declining prevalence of tobacco among young people (20).

The status of country development emerged as a pivotal determinant of the lung cancer burden. Although there was an absence of a clear correlation between the lung cancer burden and the SDI, the negligible burden stemming from the low diagnosis rates in low-SDI countries could not be ignored. The heterogeneity in the lung cancer burden among countries with SDI levels falling between 0.45 and 0.75 could be attributed to substantial differences in their economic conditions and prevention programs (21).

From 2010 to 2022, there was a gradual and sustained escalation in the number of registered lung cancer trials, particularly those that designated PROs as outcomes and utilizing explicitly specified PRO instruments. During the study period, 24.48% (952/3889) of trials conducted in WPRO mentioned the use of PROs. Among PRO-using trials, 17.75% (169/952) designated PRO instruments as primary outcomes, 67.86% (646/952) as secondary outcomes, and 17.39% (137/952) as coprimary outcomes. Meanwhile, a considerable proportion of the included trials (75.52%, 2937/3889) overlooked incorporating the PRO evaluation of lung cancer patients. Given that single-arm trials constitute more than half of all trials, it is noteworthy that the single-arm design makes it difficult to demonstrate the value of PROs in the absence of a control group, thereby discouraging the application. Within PROs trails, phase 2 trials constituted the largest proportion (249/952, 26.16%). The exclusion of PROs from early-stage trials may hinder a comprehensive assessment of the impact of the intervention on patient-reported quality of life (22). The high attrition rate in early-phase trials and the primary focus on initial safety and dosing may be barriers that suppress the utilization of PROs. Capturing all symptom reports in early phase trials is critical for improving safety, which can be facilitated by employing online system to ensure the prompt clinical interpretation of PROs (23). Revision of existing methodological guidelines is recommended to facilitate PRO inclusion in early-phase trials (24). Of all the PRO-related trials, 27.00% (257) trials used only 1 specified PRO instrument, 50.00% (476) trials used ≤4 specified PRO instruments. Trials employing an excessive number of PRO instruments often resulted in assessment that were neither patient-friendly nor feasible within the clinical practice constraints, impacting reliability (25). While using a limited number of PRO instruments would expedite the identification of individuals at an elevated risk for poor quality of life and a high symptom burden (26), this approach fell short of providing a comprehensive view of an individual’s overall health status (27). Therefore, further research could focus on developing condition-specific PRO instruments, and discovering further correlations among the PRO instruments to facilitate a more profound comprehension of PROs and simplify their implementation (28). Open-label trials comprised the largest segment for both PRO-related trials and overall trials population. Prior research has supported the validity of PRO results derived from open-label RCTs (29). Although open-label designs are feasible in certain condition, the resulting expectation bias and the inherent subjectivity of PROs cannot be overlooked. Surprisingly, minimal data indicate the presence of open-label bias regarding PRO measures in cancer clinical trials (30), yet the importance of randomization is evident. The prerequisites of randomization and blinding pose significant challenges in interventional single-arm trials, making it imperative to meticulously weigh potential risks and benefits, and to accord particular attention to the design, analysis, reporting and interpretation of PROs (31).

This study analyzed the frequency of the PRO instruments in different classification of outcomes and found that the EORTC QLQ-C30 and the EORTC QLQ-LC13 were the most commonly used in PRO-related trials. Meanwhile, these instruments maintained their prominence in trials where PROs were set as secondary and coprimary outcomes. The EORTC QLQ-C30 and the EORTC QLQ-LC13 were consistently prevalent. The EORTC QLQ-C30 includes 30 questions encompassing five domains related to cancer patients’ functioning, symptoms, financial difficulties and quality of life (32). The EORTC QLQ-LC13, often used in conjunction with the EORTC QLQ-C30, covers 13 typical lung cancer symptoms, including cough, pain, dyspnea, sore mouth, peripheral neuropathy and hair loss (33). Give the significant burden of lung cancer, PROs based on the above dimensions aid healthcare professionals and patients in making balanced decisions regarding treatment risks and benefits (34). For primary outcomes, the VAS and NRS, both single-item scales, emerged as the most commonly used instruments. Lung cancer significantly impacts patients’ quality of life, with pain being a significant concern (35), exacerbated by current therapeutic approaches (36, 37). The VAS has been recommended for pain assessment due to its reliability, while the NRS has been widely used because of its comprehensibility and ease of completion in measuring chronic pain (38). Studies have reported that the NRS surpasses the VAS in accuracy and ease of use for pain assessment, suggesting that researchers should select PRO instruments based on actual conditions (39, 40). Additionally, a significant proportion of trials employed specific PRO instruments such as the FACT-L, LCSS and SGRQ, although instruments assessing single symptoms were still in use, suggesting the recognition of the condition-specific PRO instruments for lung cancer in clinical trials.

Our study also examined the distribution and the design of the PRO-related trials in the WPRO. All countries with a high SDI, including South Korea, Japan, Singapore, New Zealand and Australia, have all embarked on PROs-related trials. Among high-middle SDI bracket, only Malaysia and China have ventured into such trials, while Vietnam stood alone as the sole middle SDI country to have conducted trials. It is notable that trials pertaining to PRO are scarce in low-middle and low SDI countries. The variation in the number of PROs-related trials held in different countries roughly aligns with prevalent and incidence of cases, albeit influenced by economic status, population demographics and medical resources (41). It would be beneficial for a broader range of low-middle and low SDI countries to engage in clinical trials using PROs. Additionally, for countries that have already embarked on PRO-related trials, establishing reference values for PRO instruments within the general population would elevate the quality of outcomes (42). Tailored strategies for promoting PROs must be designed according to SDI levels and existing foundations. In high-SDI countries, continued efforts should focus on validating and updating PRO instruments. For instance, neither the EORTC QLQ-C30 nor its lung cancer module QLQ-LC13 includes assessment of acneiform rash, a common side effect. While the EORTC QLQ-LC29 addressing this gap, it still lacks validation (43, 44). In low-SDI countries, simplified tools with fewer items may be adopted to enhance feasibility. Furthermore, given the financial implications of trials with strict methodological designs, integrating PROs into real-world studies presents a more applicable approach and could help alleviate financial constraints (45).

### Limitations

This study is not devoid of limitations. Firstly, given the possibility that registries may not cover the population in specific regions, ensuring data reliability is crucial for the GBD estimates and the analysis of PROs in clinical trials. Secondly, the reliance of proxy-reported outcomes was discouraged, rising concerns about children’s ability to provide reliable responses. Consequently, the exclusion of trials with children introduced a potential bias. Lastly, it has been observed that some registered trials lack complete information updates, and that recruitment has yet to commenced for some trials.

## Conclusion

This study examined the burden of lung cancer and the application of PROs in clinical trials pertaining to lung cancer in the WPRO. The results revealed the substantial burden of lung cancer in the region, highlighting considerable disparities among different countries. Notably, PRO instruments were employed in less than a quarter of lung cancer clinical trials, with only a scarcity of trials leveraging condition-specific PRO instruments. It is imperative for future research endeavors to advance standardized, condition-specific PRO instruments and explore the association among PRO instruments to streamline their application. Meanwhile, there is a pressing need to augment investment in PRO research in low SDI countries.

## Data Availability

Publicly available datasets were analyzed in this study. This data can be found at: https://ghdx.healthdata.org/.

## Glossary

ASDR: Age-standardized DALYs rate
ASIR: Age-standardized incidence rate
ASMR: Age-standardized mortality rate
ASRs: Age-standardized rates
BFI: Brief fatigue inventory
CFS: Cancer fatigue scale
DALYs: Disability-adjusted life years
EQ-5D: European Quality of Life-5 Dimensions
EORTC QLQ-C30: European Organization for Research and Treatment of Cancer Quality of Life Questionnaire-Core 30
EORTC QLQ-LC13: European Organization for Research and Treatment of Cancer Quality of Life Questionnaire-Lung Cancer 13
EORTC QLQ-LC43: European Organization for Research and Treatment of Cancer Quality of Life Questionnaire-Lung Cancer 43
FACT-L: Functional Assessment of Cancer Therapy-Lung
GBD: Global Burden of Disease
HADS: Hospital Anxiety Depression Scale
LCQ: Leicester Cough Questionnaire
LCSS: Lung Cancer Symptom Scale
NRS: Numeric Rating Scale
PROs: Patient-reported outcomes
PSQI: Pittsburgh Sleep Quality Index
QoL: Quality of life
QoR-40: Quality of recovery-40
SDI: Socio-Demographic Index
SF-36/SF-36v2: Short-Form 36-item Health Survey
SGRQ: St George’s Respiratory Questionnaire
UI: Uncertainty intervals
VAS: Visual Analogue Scale
WPRO: Western Pacific Region
