# Supplementary material for: Burden of lung cancer and application of patient-reported outcomes in the Western Pacific Region: a systematic analysis
Source: Front Med (Lausanne). 2025 Dec 31;12:1700961. doi: 10.3389/fmed.2025.1700961 (PMC12801346; doi:10.3389/fmed.2025.1700961)
Supplement: Supplementary file 1 [file Data_Sheet_1.pdf]

Countries, territories and areas in the WPRO
Australia, Brunei, Cambodia, China, Cook Islands, Fiji, Japan, Kiribati, Laos, Malaysia, Marshall Islands, Micronesia, Mongolia, Nauru, New Zealand, Niue, Palau, Papua New Guinea, Philippines, Samoa, Singapore, Solomon Islands, South Korea, Tonga, Tuvalu, Viet Nam.

Searching Strategy

International Clinical Trials Registry Platform - 09/01/2024

“Disease studied” = "Pulmonary Neoplasm*" OR "Neoplasm*, Lung" OR "Lung Neoplasm" OR "Neoplasm*, Pulmonary" OR "Lung Cancer*" OR "Cancer*, Lung" OR "Pulmonary Cancer*" OR "Cancer*, Pulmonary" OR "Cancer of the Lung" OR "Cancer of Lung" OR "Bronchogenic Carcinoma" OR "Lung Carcinoma" OR "Primary Lung Neoplasm" OR "Metastatic Lung Neoplasm"

“Study type” = “Interventional Study”

“Recruitment status” =ALL

“Date of registration” = “01/01/2010” To “12/31/2022”

“Phases” = ALL

Supplementary Table 1. Incidence, deaths, and disability-adjusted life years (DALYs) from lung cancer in general population in 2021 for males and females, and percentage change of age-standardized rates (ASRs) per 100000 population between 1990 and 2021 in the Western Pacific region.

	Incidence			Deaths			DALYs		
Regions	No (95% UI)	ASRs per 100000 population (95% UI)	% change in ASRs per 100000 population (95% UI)	No (95% UI)	ASRs per 100000 population (95% UI)	% change in ASRs per 100000 population (95% UI)	No (95% UI)	ASRs per 100000 population (95% UI)	% change in ASRs per 100000 population (95% UI)
Western Pacific Region	1151411.89 (957421.91 to 1359638.69)	40.17 (33.47 to 47.36)	26.50 (0.89 to 55.46)	989054.47 (821695.86 to 1164816.89)	34.70 (28.80 to 40.78)	9.04 (-13.21 to 34.60)	22393803.84 (18444329.93 to 26622164.40)	782.12 (643.87 to 927.79)	-0.51 (-21.92 to 24.67)
male	772432.75 (604466.13 to 954613.32)	58.24 (45.61 to 71.47)	20.33 (-11.99 to 56.26)	666839.19 (518156.41 to 824119.59)	51.15 (40.14 to 62.65)	4.46 (-24.04 to 36.20)	15291037.15 (11710773.50 to 19188149.97)	1118.19 (861.26 to 1396.08)	-3.81 (-31.34 to 29.10)
female	378979.142 (310763.92 to 456403.75)	24.95 (20.49 to 30.07)	38.19 (7.56 to 75.81)	322215.28(263985.78 to 387644.44)	21.10 (17.28 to 25.41)	16.96 (-8.84 to 48.58)	7102766.69 (5772034.95 to 8618148.47)	479.73 (388.89 to 582.77)	6.45 (-18.16 to 39.27)
Australia	13774.86 (12303.28 to 15149.03)	30.05 (26.98 to 32.85)	-20.19 (-27.51 to -12.39)	10246.23 (9132.27 to 11289.55)	21.90 (19.71 to 24.01)	-32.77 (-39.12 to -25.94)	208319.83 (189117.78 to 226400.12)	479.78 (438.61 to 520.37)	-38.90 (-44.06 to -33.26)
Brunei	83.68 (66.92 to 100.17)	26.18 (21.03 to 31.43)	-31.29 (-49.73 to -5.20)	79.68 (63.82 to 95.92)	26.36 (21.18 to 31.79)	-34.44 (-51.82 to -10.00)	2079.19 (1667.96 to 2506.59)	570.91 (457.27 to 687.61)	-36.62 (-53.24 to -12.47)
Cambodia	2901.55 (2063.60 to 3810.68)	23.57 (16.86 to 30.54)	3.12 (-28.45 to 46.10)	3022.17 (2174.70 to 3943.99)	25.30 (18.09 to 32.69)	3.07 (-28.42 to 46.71)	80692.53 (57461.55 to 105564.54)	609.62 (438.48 to 792.75)	-1.94 (-31.75 to 40.11)
China	934704.06 (750040.14 to 1136937.93)	44.01 (35.45 to 53.35)	32.92 (1.21 to 71.31)	814363.76 (652636.22 to 987794.68)	38.98 (31.40 to 47.06)	12.21 (-14.39 to 45.16)	18920202.62 (15100681.28 to 23111519.31)	878.24 (703.53 to 1068.71)	1.70 (-23.41 to 33.27)
Cook Islands	7.33 (5.82 to 8.87)	27.95 (22.15 to 33.77)	-15.72 (-40.72 to 12.55)	7.53 (6.00 to 9.10)	28.74 (22.91 to 34.81)	-20.19 (-43.78 to 6.62)	171.42 (136.01 to 209.79)	662.01 (522.43 to 813.08)	-20.49 (-44.46 to 6.62)
Fiji	69.30 (52.75 to 91.14)	9.24 (7.07 to 11.88)	-9.66 (-37.05 to 25.79)	72.15 (54.30 to 93.57)	9.98 (7.61 to 12.66)	-9.70 (-36.88 to 24.48)	1928.83 (1457.25 to 2515.05)	236.88 (178.74 to 307.92)	-12.66 (-40.90 to 24.72)

Regions	Incidence			Deaths			DALYs		
	No (95% UI)	ASRs per 100000 population (95% UI)	% change in ASRs per 100000 population (95% UI)	No (95% UI)	ASRs per 100000 population (95% UI)	% change in ASRs per 100000 population (95% UI)	No (95% UI)	ASRs per 100000 population (95% UI)	% change in ASRs per 100000 population (95% UI)
Japan	121731.08 (105281.87 to 131198.21)	30.24 (27.16 to 32.13)	-1.91 (-8.55 to 2.68)	92118.65 (78911.56 to 98959.47)	21.33 (19.06 to 22.57)	-15.33 (-20.55 to -11.96)	1489327.32 (1328623.09 to 1576187.52)	420.77 (387.54 to 440.11)	-23.61 (-27.49 to -20.95)
Kiribati	11.27 (8.25 to 14.99)	15.59 (11.68 to 20.48)	14.49 (-18.41 to 58.35)	11.64 (8.53 to 15.62)	16.81 (12.64 to 21.98)	14.55 (-17.95 to 58.48)	345.10 (249.58 to 466.57)	425.04 (311.20 to 568.05)	13.57 (-20.53 to 58.66)
Laos	929.06 (676.20 to 1264.12)	20.48 (15.00 to 27.64)	-10.94 (-44.59 to 34.78)	970.23 (708.82 to 1329.94)	22.10 (16.15 to 29.76)	-10.12 (-44.52 to 34.24)	26550.44 (19218.57 to 36992.15)	534.87 (389.56 to 739.48)	-15.78 (-48.49 to 27.78)
Malaysia	4953.55 (4163.53 to 5743.09)	17.49 (14.64 to 20.29)	11.75 (-18.53 to 43.00)	5050.89 (4229.03 to 5858.11)	18.22 (15.18 to 21.18)	8.80 (-20.78 to 39.99)	129683.72 (109215.79 to 150462.38)	437.43 (367.85 to 508.56)	6.08 (-21.98 to 34.87)
Marshall Islands	9.33 (5.80 to 13.47)	27.42 (17.65 to 39.15)	18.56 (-17.68 to 70.29)	9.56 (6.00 to 13.83)	29.63 (19.25 to 42.13)	18.83 (-16.97 to 70.36)	282.29 (174.04 to 411.39)	725.77 (458.45 to 1045.53)	16.34 (-21.04 to 69.71)
Micronesia	21.44 (15.13 to 29.48)	28.96 (20.68 to 39.69)	11.08 (-21.21 to 50.81)	21.99 (15.57 to 30.32)	30.95 (22.32 to 42.17)	10.29 (-21.17 to 49.01)	634.82 (436.06 to 871.67)	775.27 (547.89 to 1058.12)	9.34 (-25.17 to 50.69)
Mongolia	504.32 (385.94 to 654.64)	21.90 (16.72 to 28.47)	-28.08 (-48.90 to 2.69)	514.00 (399.69 to 665.95)	23.12 (17.96 to 30.05)	-28.45 (-49.35 to 2.71)	14685.69 (11416.31 to 18793.65)	576.78 (448.38 to 742.75)	-30.31 (-50.69 to -0.61)
Nauru	2.21 (1.17 to 2.86)	38.36 (20.90 to 49.01)	-3.17 (-30.80 to 38.72)	2.26 (1.21 to 2.90)	40.86 (22.14 to 52.08)	-4.56 (-31.31 to 36.46)	66.31 (35.47 to 86.40)	1032.45 (551.47 to 1329.50)	-2.87 (-31.50 to 41.14)
New Zealand	2602.62 (2338.86 to 2866.02)	30.36 (27.42 to 33.30)	-22.19 (-30.26 to -12.49)	2014.24 (1819.76 to 2205.89)	23.29 (21.14 to 25.47)	-35.02 (-41.64 to -27.52)	42976.68 (39450.84 to 46784.45)	524.69 (480.55 to 570.94)	-39.48 (-45.80 to -32.38)
Niue	0.60 (0.45 to 0.77)	27.42 (20.82 to 35.29)	23.84 (-13.35 to 67.90)	0.63 (0.48 to 0.81)	29.28 (22.28 to 37.50)	22.44 (-13.81 to 65.66)	15.00 (11.28 to 19.46)	684.14 (513.82 to 892.01)	18.63 (-18.02 to 61.07)
Palau	9.53 (7.44 to 11.86)	44.74 (35.53 to 55.18)	-3.13 (-28.87 to 32.04)	9.79 (7.59 to 12.20)	48.28 (38.23 to 59.49)	-3.95 (-29.35 to 29.72)	259.57 (198.47 to 324.99)	1106.68 (865.22 to 1379.51)	-6.57 (-32.34 to 29.05)

Regions	Incidence			Deaths			DALYs		
	No (95% UI)	ASRs per 100000 population (95% UI)	% change in ASRs per 100000 population (95% UI)	No (95% UI)	ASRs per 100000 population (95% UI)	% change in ASRs per 100000 population (95% UI)	No (95% UI)	ASRs per 100000 population (95% UI)	% change in ASRs per 100000 population (95% UI)
Papua New Guinea	822.74 (544.73 to 1272.58)	17.22 (11.36 to 26.42)	13.17 (-23.41 to 66.83)	854.38 (559.53 to 1331.64)	18.74 (12.20 to 29.17)	13.62 (-23.15 to 68.52)	24354.61 (15862.19 to 38261.26)	445.36 (291.55 to 694.06)	10.75 (-25.58 to 67.64)
Philippines	13133.10 (10827.62 to 15687.41)	15.87 (13.18 to 18.88)	-5.79 (-27.48 to 16.42)	13555.02 (11193.53 to 16172.93)	16.82 (14.01 to 19.98)	-8.13 (-29.57 to 13.44)	373370.68 (305719.42 to 447190.52)	420.94 (346.12 to 503.58)	-6.15 (-28.30 to 16.88)
Samoa	11.96 (8.90 to 15.54)	8.28 (6.22 to 10.62)	-0.59 (-26.70 to 30.48)	12.39 (9.28 to 16.03)	8.75 (6.57 to 11.28)	-1.98 (-27.47 to 29.54)	329.80 (240.16 to 434.08)	217.22 (159.93 to 284.09)	-1.60 (-29.43 to 30.53)
Singapore	1859.33 (1665.50 to 2079.26)	21.95 (19.66 to 24.37)	-38.62 (-46.63 to -30.98)	1359.15 (1220.56 to 1516.81)	16.19 (14.52 to 18.03)	-52.98 (-58.73 to -47.22)	28086.17 (25230.89 to 31479.44)	326.14 (292.32 to 364.90)	-58.62 (-63.84 to -53.43)
Solomon Islands	71.06 (52.98 to 96.59)	19.54 (14.95 to 26.27)	2.82 (-31.31 to 60.19)	72.17 (53.91 to 98.08)	20.66 (15.79 to 27.69)	1.70 (-31.68 to 57.39)	2272.55 (1666.47 to 3118.98)	555.52 (414.31 to 756.37)	4.16 (-32.31 to 71.04)
South Korea	30951.76 (25826.93 to 36484.47)	32.50 (27.16 to 38.38)	20.82 (-2.75 to 49.16)	22630.53 (18701.04 to 26778.23)	23.85 (19.66 to 28.24)	-11.11 (-28.79 to 10.59)	439871.47 (370419.16 to 515239.41)	464.44 (391.27 to 544.82)	-31.04 (-44.04 to -14.64)
Tonga	21.96 (17.02 to 27.87)	27.71 (21.53 to 35.00)	4.92 (-26.20 to 48.16)	23.38 (18.10 to 29.49)	29.74 (23.11 to 37.44)	3.49 (-26.85 to 43.49)	565.71 (431.29 to 724.34)	696.25 (532.44 to 888.02)	3.13 (-28.10 to 45.24)
Tuvalu	2.42 (1.88 to 3.20)	23.18 (18.06 to 30.51)	11.83 (-16.89 to 47.77)	2.54 (1.98 to 3.33)	24.89 (19.41 to 32.71)	11.44 (-17.41 to 48.32)	66.67 (51.15 to 89.01)	609.34 (468.08 to 813.82)	8.74 (-20.40 to 45.70)
Vanuatu	29.18 (19.42 to 45.71)	17.21 (11.62 to 26.82)	1.89 (-22.99 to 37.52)	30.35 (20.25 to 47.29)	18.65 (12.62 to 29.01)	1.42 (-23.15 to 36.00)	857.59 (563.11 to 1352.13)	453.81 (301.30 to 706.48)	1.85 (-24.90 to 39.40)
Viet Nam	22192.59 (16587.57 to 27516.18)	21.56 (16.23 to 26.42)	15.56 (-19.10 to 60.28)	21999.16 (16349.80 to 27135.98)	21.83 (16.36 to 26.56)	11.18 (-21.15 to 52.10)	605807.22 (446380.84 to 761375.88)	561.81 (416.24 to 698.01)	8.99 (-25.01 to 54.66)

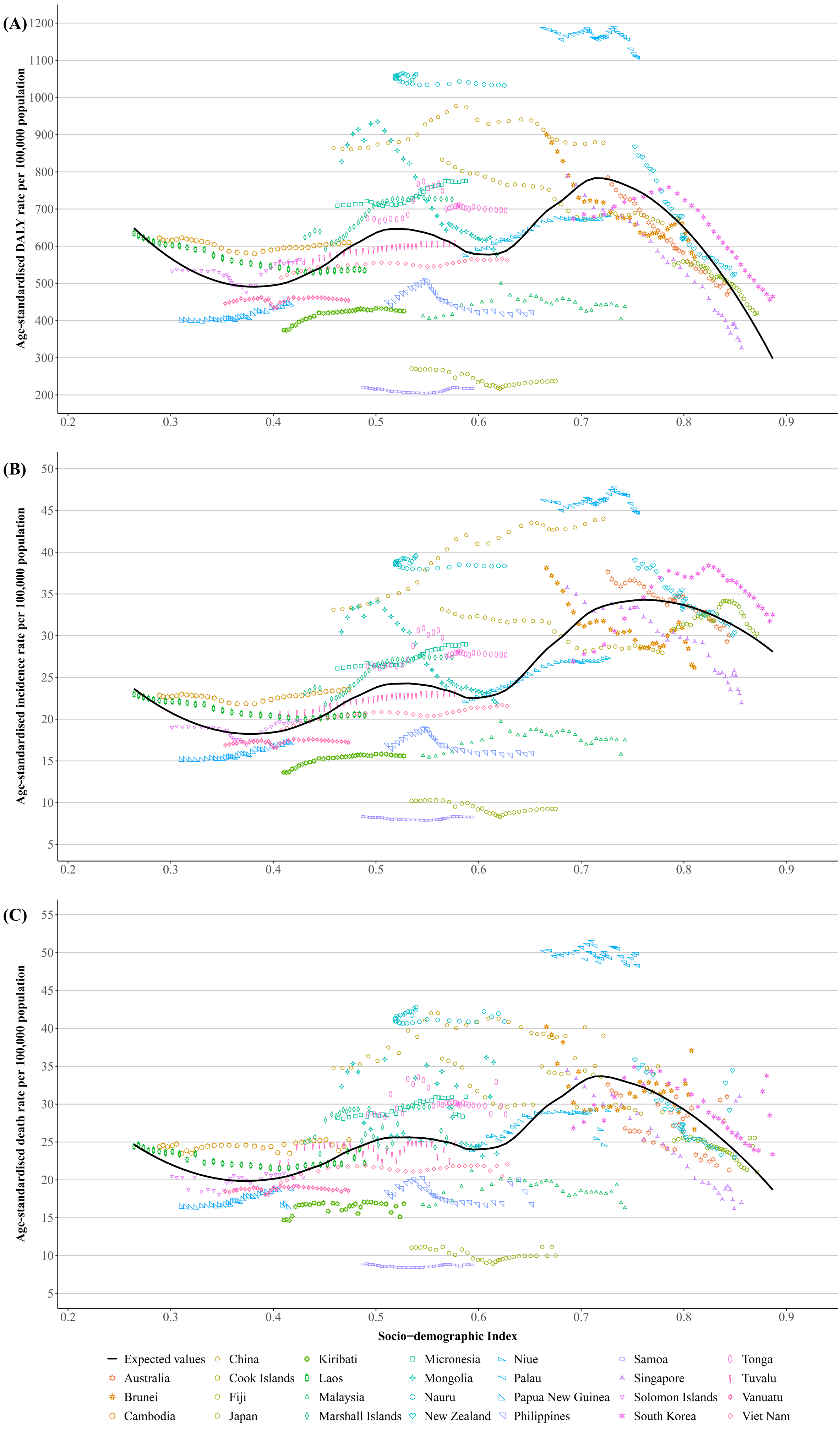

(A)
Age-standardised DALY rate per 100,000 population
(B)
Age-standardised incidence rate per 100,000 population
(C)
Age-standardised death rate per 100,000 population
Socio−demographic Index
Expected values
Australia
Brunei
Cambodia
China
Cook Islands
Fiji
Japan
Kiribati
Laos
Malaysia
Marshall Islands
Micronesia
Mongolia
Nauru
New Zealand
Niue
Palau
Papua New Guinea
Philippines
Samoa
Singapore
Solomon Islands
South Korea
Tonga
Tuvalu
Vanuatu
Viet Nam

Supplementary Figure 1. Age-standardized rate of disability-adjusted life year (DALY) (A), incidence (B) and death (C) from lung cancer per 100,000 population for 27 countries in the Western Pacific region by socio-demographic index, 1990-2021.

Black line represents expected values based on socio-demographic index and disease rates in all countries.
